# Clinical outcomes based on multigene profiling in metastatic breast cancer patients

**DOI:** 10.18632/oncotarget.12987

**Published:** 2016-10-28

**Authors:** Reva K. Basho, Debora de Melo Gagliato, Naoto T. Ueno, Chetna Wathoo, Huiqin Chen, Maryam Shariati, Caimiao Wei, Ricardo H. Alvarez, Stacy L. Moulder, Aysegul A. Sahin, Sinchita Roy-Chowdhuri, Mariana Chavez-MacGregor, Jennifer K. Litton, Vincent Valero, Raja Luthra, Jia Zeng, Kenna R. Shaw, John Mendelsohn, Gordon B. Mills, Debu Tripathy, Funda Meric-Bernstam

**Affiliations:** ^1^ Division of Cancer Medicine, The University of Texas MD Anderson Cancer Center, Houston, TX, USA; ^2^ Breast Medical Oncology, The University of Texas MD Anderson Cancer Center, Houston, TX, USA; ^3^ Sheikh Khalifa Bin Zayed Al Nahyan Institute for Personalized Cancer Therapy, The University of Texas MD Anderson Cancer Center, Houston, TX, USA; ^4^ Department of Biostatistics, The University of Texas MD Anderson Cancer Center, Houston, TX, USA; ^5^ Investigational Cancer Therapeutics (Phase I Trials Program), The University of Texas MD Anderson Cancer Center, Houston, TX, USA; ^6^ Pfizer, Inc, New York, NY, USA; ^7^ The Cancer Treatment Centers of America, Chicago, IL, USA; ^8^ Department of Pathology, The University of Texas MD Anderson Cancer Center, Houston, TX, USA; ^9^ Health Services Research, The University of Texas MD Anderson Cancer Center, Houston, TX, USA; ^10^ Department of Genomic Medicine, The University of Texas MD Anderson Cancer Center, Houston, TX, USA; ^11^ Department of Systems Biology, The University of Texas MD Anderson Cancer Center, Houston, TX, USA; ^12^ Breast Surgical Oncology, The University of Texas MD Anderson Cancer Center, Houston, TX, USA

**Keywords:** metastatic breast cancer, genomics, TP53, PIK3CA

## Abstract

**BACKGROUND:**

Identifying the clinical impact of recurrent mutations can help define their role in cancer. Here, we identify frequent hotspot mutations in metastatic breast cancer (MBC) patients and associate them with clinical outcomes.

**PATIENTS AND METHODS:**

Hotspot mutation testing was conducted in 500 MBC patients using an 11 gene (*N* = 126) and/or 46 or 50 gene (*N* = 391) panel. Patients were stratified by hormone receptor (HR) and human epidermal growth factor 2 (HER2) status. Clinical outcomes were retrospectively collected.

**RESULTS:**

Hotspot mutations were most frequently detected in *TP53* (30%), *PIK3CA* (27%) and *AKT1* (4%). Triple-negative breast cancer (TNBC) patients had the highest incidence of *TP53* (58%) and the lowest incidence of *PIK3CA* (9%) mutations. *TP53* mutation was associated with shorter relapse-free survival (RFS) (median 22 *vs* 42months; *P* < 0.001) and overall survival (OS) from diagnosis of distant metastatic disease (median 26 *vs* 51months; *P* < 0.001). Conversely, *PIK3CA* mutation was associated with a trend towards better clinical outcomes including RFS (median 41 *vs* 30months; *P* = 0.074) and OS (52 *vs* 40months; *P* = 0.066). In HR-positive patients, *TP53* mutation was again associated with shorter RFS (median 30 *vs* 46months; *P* = 0.017) and OS (median 30 *vs* 55months; *P* = 0.001). When multivariable analysis was performed for RFS and OS, *TP53* but not *PIK3CA* mutation remained a significant predictor of outcomes in the overall cohort and in HR-positive patients.

**CONCLUSIONS:**

Clinical hotspot sequencing identifies potentially actionable mutations. In this cohort, *TP53* mutation was associated with worse clinical outcomes, while *PIK3CA* mutation did not remain a significant predictor of outcomes after multivariable analysis.

## INTRODUCTION

Breast cancer is the most frequently diagnosed cancer and is the second leading cause of cancer death among females.[1, 2] Despite advances in detection and treatment, 20-30% of patients with early-stage breast cancer will become metastatic.[3] The development of targeted therapy for HR-positive and HER2-overexpressing MBC has significantly improved outcomes in these subsets.[4-9] However, upon development of resistance to these therapies and in the setting of TNBC, cytotoxic chemotherapy remains the backbone of treatment, and long-term outcomes remain poor.[10]

Understanding the genomic drivers of cancer growth is essential to developing new therapies. Further, as tumors become resistant to administered therapies, tumor heterogeneity develops, making genomically-informed therapy even more relevant to the constantly changing landscape of advanced disease. The emerging capability of sequencing multiple cancer-related genes in a tissue-sparing and cost-effective manner and the development of novel targeted therapeutics has made genomically-informed therapy a reality. However, although molecular testing is now routinely performed in many patients with advanced cancer, the use of this knowledge to guide therapy is widely accepted for only a few alterations in specific tumor types. Thus, we need more comprehensive knowledge of genomic changes and their clinical implications.

Through sequencing, we have learned that *TP53* and *PIK3CA* are the most frequently mutated genes in breast cancer.[11] However, the clinical implications of these mutations are not well defined. Because *TP53* is a multifunctional protein, some studies report improved outcomes with mutations while others report the opposite.[12] Similarly, studies report varying outcomes with mutations in *PIK3CA*.[13-15] The implications of various mutations become even more complex when specific subsets of breast cancer are evaluated. Large patient series with available genomic and clinical outcome data are needed to better understand implications of aberrations in specific genes. Thus, we activated an enterprise-level genomic screening effort. In this study, we reviewed the results of hotspot molecular testing by next-generation sequencing (NGS) of commonly mutated genes in the tumors of 500 patients with MBC. We then correlated the most frequently encountered hotspot mutations with clinical and pathologic characteristics and clinical outcomes to better understand their clinical implications. In this cohort of MBC patients, *TP53*, the most frequently encountered hotspot mutation, was associated with worse clinical outcomes, while *PIK3CA*, the second most frequently encountered hotspot mutation, did not remain a significant predictor of outcomes after multivariable analysis.

## RESULTS

A total of 605 samples from 500 MBC patients were collected. The primary tumor was tested in most patients (290). Two samples were tested in 103 patients. Of these, the majority of patients (89) had the primary and a metastatic site analyzed. One patient had 3 samples tested (primary, synchronous axillary lymph node, metachronous metastatic disease). Patients who had more than one sample tested were considered mutants for a specific gene if mutations were encountered in any of the samples tested. Sequenom was used exclusively in 109 patients and a 46 or 50 gene Ampliseq Ion Torrent Assay was used exclusively in 374 patients. The genes assessed by each assay are listed is Supplementary Table 1.

Seventeen patients underwent analysis by both Sequenom and the 46 gene assay. The nine common genes between these two testing methods and their mutation status in each patient are shown in Supplementary Figure 1. There was mutational disconcordance between the two testing platforms in three of the seventeen cases; however in all of these patients, different sites were analyzed by Sequenom and the Ampliseq 46 panel. In addition, the time interval between obtaining samples from the primary and metastatic tumors in these three patients may be relevant as it is possible that mutations can accumulate within a tumor over time. In one case, the metastatic specimen was collected two years after the primary sample.

Supplementary Figure 2 illustrates the mutation status of genes between primary tumors and corresponding metastases in the 89 patients that had both sites analyzed. Matched primary and metastatic tumors demonstrated an overall concordance rate of 74% (66/89). The aberrant genes associated with primary-metastasis discordance were identified predominantly in metastatic tumors, with *PIK3CA* and *TP53* being the most frequent discordant genes. There were four cases with gains and one with a loss of *TP53* mutation in metastatic/recurrent tumors compared to the primary. There were five cases with gains and six with losses in *PIK3CA* mutation. There were also four cases with gains and one with a loss in *MAPK* pathway mutation.

### Genomic alterations

**Figure 1:**
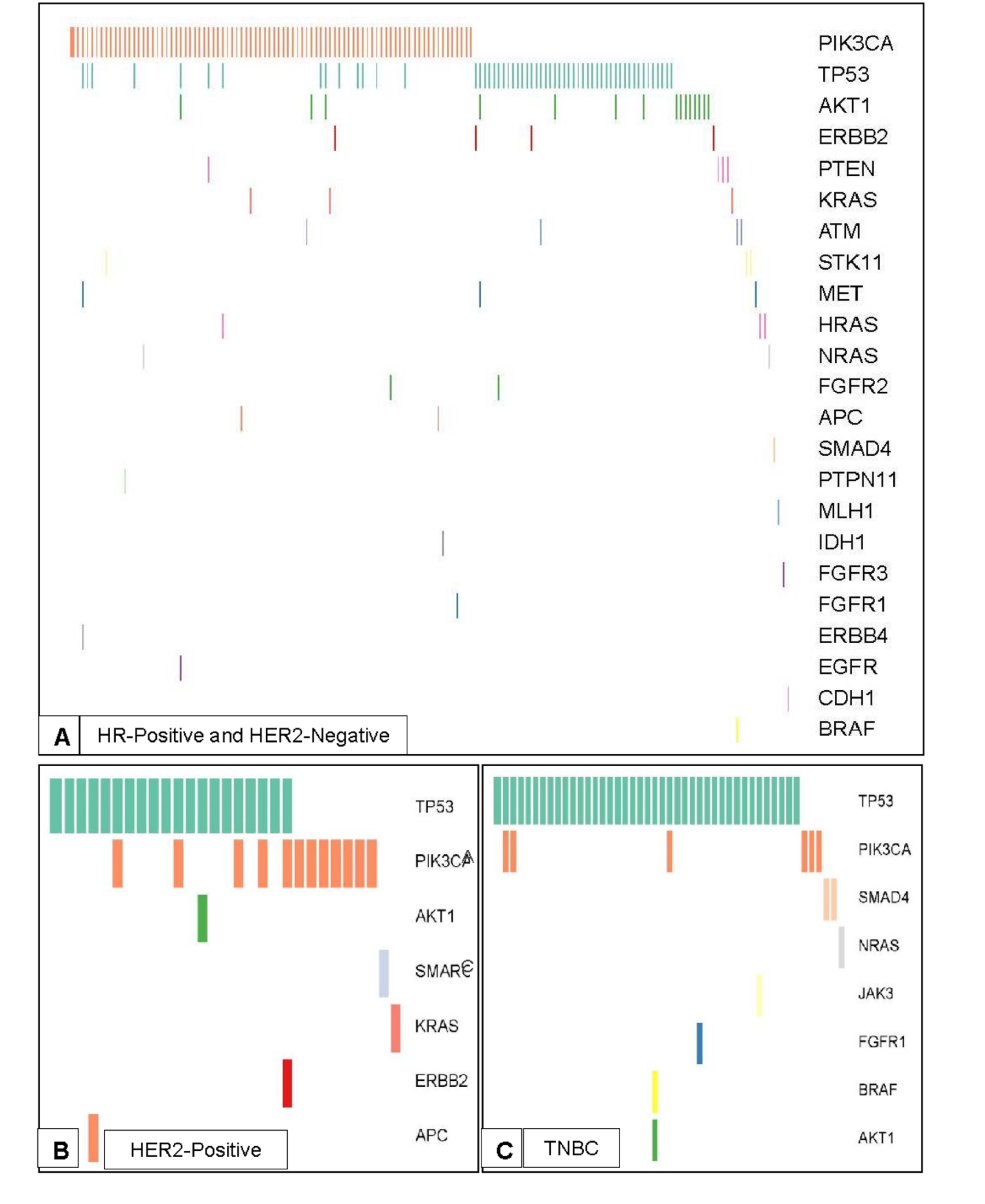
Spectrum of Mutations Detected by the 46 or 50 Gene Ampliseq Ion Torrent Assay Spectrum of mutations and co-mutations detected by the 46 or 50 gene Ampliseq Ion Torrent Assay in the three breast cancer subtypes. **A.** Spectrum of mutations in patients with HR-positive and HER2-negative disease (*N* = 154). **B.** Spectrum of mutations in patients with HER2-positive disease (*N* = 29). **C.** Spectrum of mutations in patients with TNBC (*N* = 47).

Genomic alterations that were predicted to be germline variants based on an informatics algorithm that assessed allelic frequency of mutations in tumor samples were excluded (Supplementary Table 2). The most frequently mutated genes were *TP53* (29.9%), *PIK3CA* (27.2%) and *AKT1* (4.0%). Alterations were also detected in other genes including *ERBB2* (1.3%), *KRAS* (1%), *PTEN* (1%), *HRAS* (0.8%), *SMAD4* (0.8%), *ATM* (0.8%), *NRAS* (0.6%), *FGFR2* (0.5%), *BRAF* (0.4%), *IDH1* (0.4%), *APC* (0.5%), *CDH1* (0.3%), *EGFR* (0.3%), *ERBB4* (0.3%), *FGFR1* (0.3%), *FGFR3* (0.3%), *JAK3* (0.3%), *MLH1* (0.3%), *PTPN11* (0.3%), *SMARCB1* (0.3%), *STK11* (0.3%), and *MET* (0.4 %). Alterations seen by the 46 or 50 gene panels in replace each of the subtypes with each subset of breast cancer are presented in Figure 1.

### Correlation of frequent mutations with clinical and pathologic characteristics

For clinical analysis, four of the 500 patients were excluded because they did not start systemic therapy at the time of diagnosis. Nine additional patients were excluded because they developed contralateral breast cancer prior to metastatic disease. After exclusion, 343 patients (70.4%) had HR-positive and HER2-negative breast cancer, 55 (11.3%) had HER2-positive disease regardless of HR status and 89 (18.3%) had TNBC. Table 1 summarizes the patient characteristics, overall and by mutation in *TP53*, *PIK3CA* or *AKT1*. Patients with TNBC had a higher rate of *TP53* mutation (58.3%) than HER2-positive (42.4%) and HR-positive (21.7%) patients. Conversely, *PIK3CA* mutation rate was lower in TNBC (9.0%) than HER2-positive (30.9%) and HR-positive (31.2%) disease. All 19 *AKT1* mutated cases were HER2-negative, and 18 out of 19 cases were HR-positive. In patients with grade III tumors, the incidence of *TP53* mutation (39.5%) was higher than the incidence of *PIK3CA* mutation (18.9%) or *AKT1* mutation (2.2%). Similarly, patients with intracranial disease as their first site of distant disease had a higher incidence of *TP53* mutation (57.1%) compared to *PIK3CA* mutation (14.3%) or *AKT1* mutation (0%). There were no significant differences in the mutation rates based on metastatic site tested.

**Table 1:** Summary of Patient and Clinical Characteristics by Most Frequent Mutations

	Overall	TP53 Wild Type	*TP53* Mutated	P Value	*PIK3CA* Wild Type	*PIK3CA* Mutated	P Value	*AKT1* Wild Type	*AKT1* Mutated	P Value
	N=487	N=265	N=116		N=355	N=132		N=468	N=19	
**Median Age (Range)**	47 (23-74)	46 (23-74)	48 (26-74)	0.38	47 (23-74)	48 (26-74)	0.14	47 (23-74)	40 (31-63)	0.09
**Race**				0.99			0.12			0.68
White	366	204 (70.3)	90 (30.6)		257 (70.2)	109 (29.8)		351 (95.9)	15 (4.1)	
Black	45	23 (71.9)	9 (28.1)		35 (87.8)	10 (22.2)		44 (97.8)	1 (2.2)	
Hispanic	56	25 (69.4)	11 (30.6)		46 (82.1)	10 (17.9)		53 (94.6)	3 (5.4)	
Other	20	13 (68.4)	6 (31.6)		17 (85.0)	3 (15.0)		20 (100.0)	0 (0.0)	
**Gender**				0.81			0.30			0.69
Female	483	262 (69.5)	115 (30.5)		353 (73.1)	130 (26.9)		464 (96.1)	19 (3.9)	
Male	4	3 (75.0)	1 (25.0)		2 (50.0)	2 (50.0)		4 (100.0)	0 (0.0)	
**Subtype**				<0.01			<0.01			0.06
HR-positive, HER2-negative	343	216 (78.3)	60 (21.7)		236 (68.8)	107 (31.2)		325 (94.8)	18 (5.2)	
HER2-positive	55	19 (57.6)	14 (42.4)		38 (69.1)	17 (30.9)		55 (100.0)	0 (0.0)	
TNBC	89	30 (41.7)	42 (58.3)		81 (91.0)	8 (9.0)		88 (98.9)	1 (1.1)	
**Stage at Diagnosis**				0.32			0.79			0.42
0	4	2 (66.7)	1 (33.3)		3 (75.0)	1 (25.0)		4 (100.0)	0 (0.0)	
I	62	37 (69.8)	16 (30.2)		44 (71.0)	18 (29.0)		59 (95.2)	3 (4.8)	
II	181	97 (66.4)	49 (33.5)		127 (70.2)	54 (29.8)		173 (95.6)	8 (4.4)	
III	128	70 (66.7)	35 (33.3)		98 (76.6)	30 (23.4)		121 (94.5)	7 (5.5)	
IV	111	59 (79.7)	15 (20.3)		82 (73.9)	29 (26.1)		110 (99.1)	1 (0.9)	
**Tumor Histology**				0.12			0.61			0.52
Ductal	401	216 (67.3)	105 (32.7)		298 (74.3)	103 (25.7)		386 (96.3)	15 (3.7)	
Lobular	44	26 (86.7)	4 (13.3)		28 (63.6)	16 (36.4)		42 (95.5)	2 (4.5)	
Mixed Ductal/Lobular	15	10 (76.9)	3 (23.1)		10 (66.7)	5 (33.3)		14 (93.3)	1 (6.7)	
Metaplastic/Sarcomatoid	7	2 (50.0)	2 (50.0)		5 (71.4)	2 (28.6)		6 (85.7)	1 (14.3)	
Other	20	11 (84.6)	2 (15.4)		14 (70.0)	6 (30.0)		20 (100.0)	0 (0.0)	
**Tumor Grade**				<0.01			<0.01			0.04
I	22	16 (94.1)	1 (5.9)		10 (45.5)	12 (54.5)		22 (100.0)	0 (0.0)	
II	168	110 (80.3)	27 (19.7)		106 (63.1)	62 (36.9)		157 (93.5)	11 (6.5)	
III	275	127 (60.5)	83 (39.5)		223 (81.1)	52 (18.9)		269 (97.8)	6 (2.2)	
**Neoadjuvant Chemotherapy**				<0.01			0.11			0.04
Yes	156	80 (60.6)	52 (39.4)		121 (77.6)	35 (22.4)		154 (98.7)	2 (1.3)	
No	331	185 (74.3)	64 (25.7)		234 (70.7)	97 (29.3)		314 (94.9)	17 (5.1)	
**Adjuvant Chemotherapy**				0.05			0.43			0.02
Yes	341	189 (66.8)	94 (33.2)		245 (71.8)	96 (28.2)		323 (94.7)	18 (5.3)	
No	146	76 (77.6)	22 (22.4)		110 (75.3)	36 (24.7)		145 (99.3)	1 (0.7)	
**Type of Surgery**				0.07			0.83			0.92
Breast-conserving	126	61 (59.2)	42 (40.8)		91 (72.2)	35 (27.8)		120 95.2)	6 (4.8)	
Mastectomy	299	166 (71.6)	66 (28.4)		216 (72.2)	83 (27.8)		287 (96.0)	12 (4.0)	
**First Site of Metastasis**				0.01			0.19			0.15
Visceral	181	91 (64.1)	51 (35.9)		130 (71.8)	51 (28.2)		175 (96.7)	6 (3.3)	
Bone	183	102 (73.9)	36 (26.1)		126 (68.9)	57 (31.1)		176 (96.2)	7 (3.8)	
Soft Tissue	49	29 (67.4)	14 (32.6)		41 (83.7)	8 (16.3)		44 (89.8)	5 (10.2)	
Brain	14	6 (42.9)	8 (57.1)		12 (85.7)	2 (14.3)		14 (100.0)	0 (0.0)	
Multiple	60	37 (84.1)	7 (15.9)		46 (77.7)	14 (23.3)		59 (98.3)	1 (1.7)	

### Correlation of frequent mutations with patient outcomes

**Figure 2:**
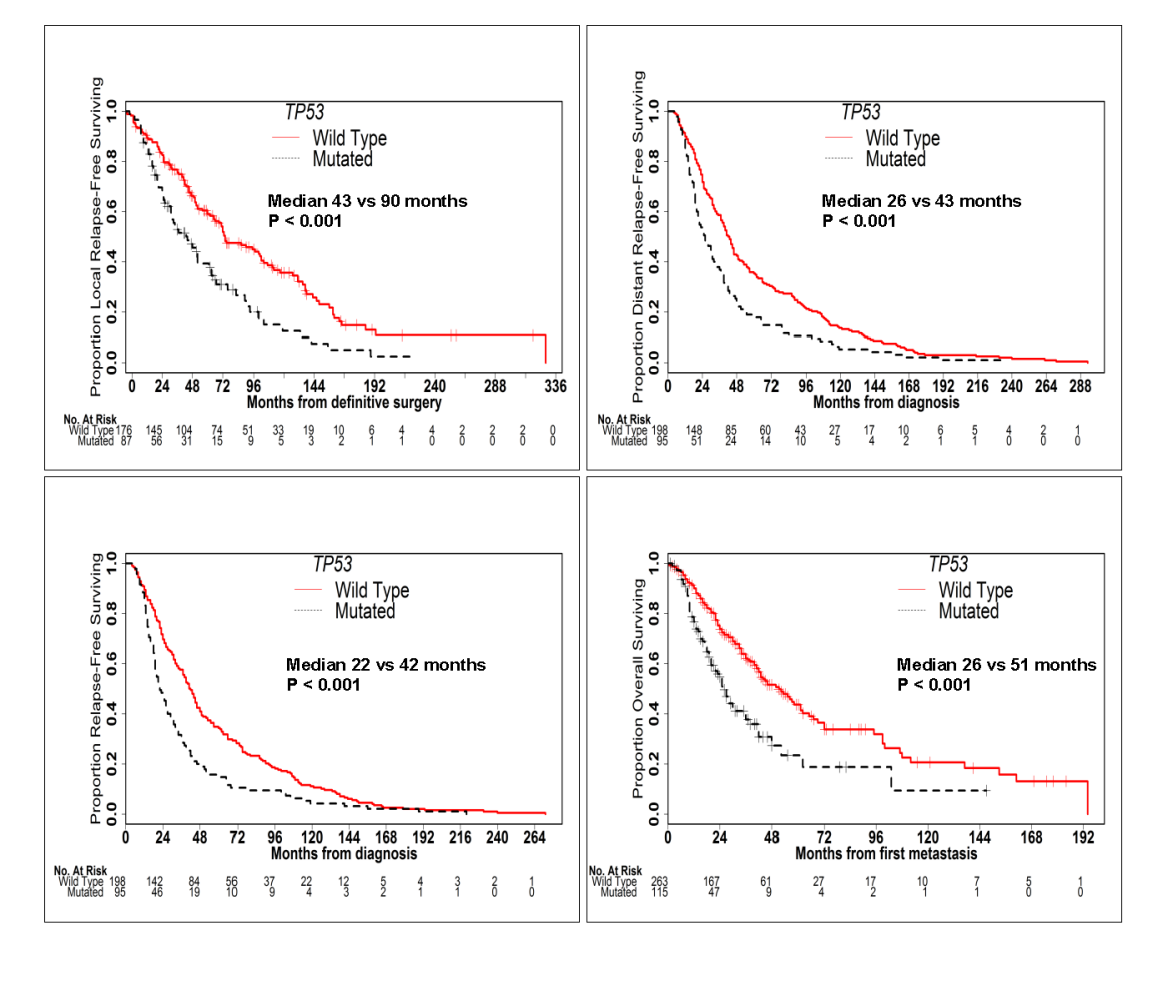
*TP53* Mutation is Associated with Worse Outcomes Kaplan-Meier curves of LRFS, DRFS, RFS and OS in patients with and without *TP53* mutations. *TP53* mutation was associated with significantly worse LRFS, DRFS, RFS and OS.

**Figure 3:**
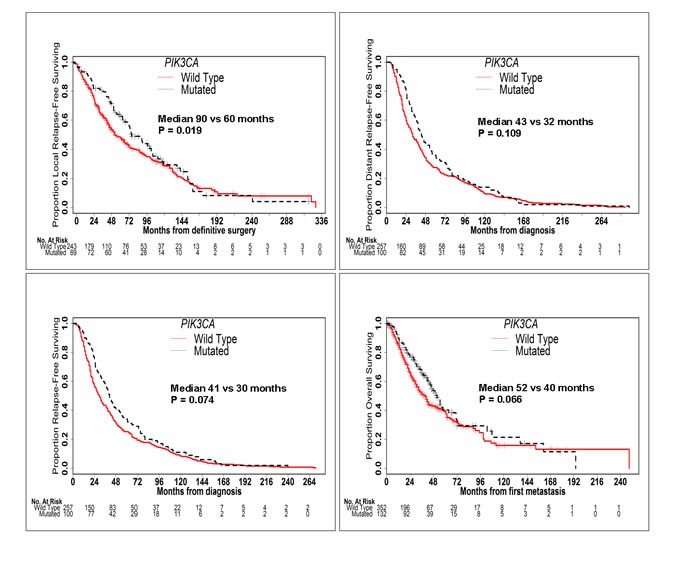
*PIK3CA* Mutation is Associated with Better Outcomes Kaplan-Meier curves of LRFS, DRFS, RFS and OS in patients with and without *PIK3CA* mutations. *PIK3CA* mutation was associated with significantly better LRFS, RFS and OS and a trend towards better DRFS.

*TP53* mutation was associated with significantly worse LRFS (median 43 *vs* 90 months; *P* < 0.001), DRFS (median 26 *vs* 43 months; *P* < 0.001), RFS (median 22 *vs* 42 months; *P* < 0.001) and OS (median 26 *vs* 51 months; *P* < 0.001) (Figure 2). Conversely, *PIK3CA* mutation was associated with significantly better LRFS (median 90 *vs* 60 months; *P* = 0.019) and a trend towards better DRFS (median 43 *vs* 32 months; *P* = 0.109), RFS (median 41 *vs* 30 months; *P* = 0.074) and OS (median 52 *vs* 40 months; *P* = 0.066) (Figure 3). When mutation in *PIK3CA* exon 9 was compared to mutation in *PIK3CA* exon 20, no significant difference was seen in RFS (41 *vs* 43 months; *P* = 0.354) or OS (53 *vs* 64 months; *P* = 0.635). *AKT1* mutation was associated with significantly better DRFS (median 66 *vs* 34 months; *P* = 0.043) and RFS (median 53 *vs* 31 months; *P* = 0.039), but not OS (median 62 *vs* 42 months; *P* = 0.211).

In HR-positive patients, *TP53* mutation was associated with significantly worse LRFS (median 61 *vs* 104 months; *P* < 0.001), DRFS (median 33 *vs* 46 months; *P* = 0.041), RFS (median 30 *vs* 46 months; *P* = 0.017) and OS (median 30 *vs* 55 months; *P* = 0.001) (Supplementary Figure 3). Conversely, presence of *PIK3CA* or *AKT1* mutation did not significantly affect outcomes in HR-positive patients. Once again, when mutation in *PIK3CA* exon 9 was compared to mutation in *PIK3CA* exon 20, no significant difference was seen in RFS (41 *vs* 44 months; *P* = 0.449) or OS (55 *vs* 50 months; *P* = 0.953). In HER2-positive patients, presence of *TP53* mutation was associated with worse RFS (median 15 *vs* 31 months; *P* = 0.016) but not OS. No other significant differences were seen in the outcomes of HER2-positive or TNBC patients with the presence of mutation in *TP53, PIK3CA* or *AKT1*.

In univariable analysis of RFS, age, clinical subtype, tumor grade, stage at diagnosis, administration of neoadjuvant or adjuvant chemotherapy and type of surgery were all significant or marginally significant predictors of outcome (Table 2). After multivariable analysis with stratification of adjuvant chemotherapy, only tumor subtype, stage at diagnosis and *TP53* mutation remained significant predictors of RFS. In HR-positive patients, grade III disease, stage II disease at diagnosis, administration of neoadjuvant chemotherapy and *TP53* mutation were significant predictors of RFS after multivariable analysis. In both the entire cohort and HR-positive patients, *PIK3CA* and *AKT1* mutations were not significant predictors of RFS after multivariable analysis.

**Table 2:** Univariable and Multivariable Analysis for RFS from Primary Cancer Diagnosis and OS from Diagnosis of Metastasis

RFS FROM PRIMARY CANCER DIAGNOSIS						
	All Patients			HR-Positive Patients		
Covariate	Univariable	Multivariable		Univariable	Multivariable	
	P Value	HR (95% CI)	P Value	P Value	HR(95%CI)	P Value
**Age**						
≤ 50	0.059	1		0.231	1	
> 50		1.28(0.98-1.66)	0.066		1.21 (0.90-1.65)	0.213
**Subtype**						
HR-positive, HER2-negative		1				
HER2-positive	<0.001	2.74 (1.59-4.72)	<0.001			
TNBC		1.58 (1.11-2.25)	0.011			
**Grade**						
I-II	<0.001	1		<0.001	1	
III		1.27 (0.97-1.65)	0.086		1.44 (1.07-1.94)	0.018
**Stage at Diagnosis**						
0-I		1			1	
II	<0.001	1.58 (1.12-2.23)	0.009	<0.001	1.73 (1.17-2.56)	0.006
III		1.99 (1.33-3.00)	0.001		1.47 (0.92-2.34)	0.110
**Neoadjuvant Chemotherapy**						
No	<0.001	1		<0.001	1	
Yes		1.22 (0.93-1.60)	0.146		1.66 (1.20-2.31)	0.003
**Surgery Type**						
Breast-Conserving	0.005	1		0.030	1	
Mastectomy		1.23 (0.91-1.66)	0.184		1.35 (0.94-1.94)	0.101
**Mutation**						
*AKT1*-mutated		0.89 (0.51-1.53)	0.663		0.92 (0.51-1.64)	0.770
*PIK3CA*-mutated		0.85 (0.65-1.13)	0.266		0.83 (0.61-1.13)	0.241
*TP53*-mutated		1.48 (1.10-1.98)	0.009		1.64 (1.15-2.33)	0.006
**OS FROM DIAGNOSIS OF METASTASIS**						
	**All Patients**			**HR-Positive Patients**		
**Covariate**	**Univariable**	**Multivariable**		**Univariable**	**Multivariable**	
	**P Value**	**HR (95% CI)**	**P Value**	**P Value**	**HR(95%CI)**	**P Value**
**Subtype**						
HR-positive, HER2-negative	<0.001	1				
HER2-positive		0.28 (0.13-0.63)	0.002			
TNBC		2.37 (1.57-3.58)	<0.001			
**Grade**						
I-II	<0.001	1		< 0.001	1	
III		1.38 (0.97-1.96)	0.072		1.50 (1.02-2.20)	0.037
**First Metastatic Site**						
Bone	0.058	1		0.168	1	
Visceral		1.21 (0.85-1.73)	0.298		1.57 (1.02-2.39)	0.038
Soft Tissue		0.63 (0.37-1.08)	0.090		0.52 (0.26-1.03)	0.064
Brain or Multiple		1.44 (0.87-2.37)	0.154		1.32 (0.69-2.53)	0.41
**Mutation**						
*AKT1*-mutated		0.61 (0.24-1.52)	0.289		0.65 (0.26-1.65)	0.367
*PIK3CA*-mutated		0.84 (0.58-1.22)	0.370		0.88 (0.59-1.32)	0.540
*TP53*-mutated		1.44 (1.00-2.07)	0.048		2.20 (1.41-3.44)	0.001

In univariable analysis of OS, clinical subtype, tumor grade, stage at diagnosis and first site of distant metastatic disease were significant or marginally significant predictors of OS (Table 3). After multivariable analysis with stratification of stage, only tumor subtype and presence of *TP53* mutation remained significant predictors. In HR-positive patients, grade III disease, visceral metastatic disease and *TP53* mutation were significant predictors of OS after multivariable analysis. Again, in both the entire cohort and HR-positive patients, *PIK3CA* and *AKT1* mutations were not significant predictors of OS after multivariable analysis.

### Analysis by therapy

TNBC patients had the worst overall TTP (median 6 months with first line therapy; median 3 months with second line therapy; and median 4 months with third line therapy), while HER2-positive patients had the best overall TTP (median 30 months with first line therapy; median 18 months with second line therapy; and median 11 months with third line therapy). In the overall patient population, targeted therapy was associated with better TTP with first (median 14) and second (median 14) line therapy. Conversely, patients treated with chemotherapy had the worst TTP with first (median 8 months) and second (median 6 months) line therapy.

*TP53* mutation was associated with significantly worse TTP with first (median 6 *vs* 13 months; *P* = 0.001) and second line therapy (median 4 *vs* 8 months; P = 0.018) (Supplementary Figure 4). In patients treated with chemotherapy, *TP53* mutation was associated with significantly worse TTP with first (median 6 *vs* 8 months; *P* = 0.046) and second (3 *vs* 8 months; *P* = 0.003) line therapy. However, *PIK3CA* and *AKT1* mutations were not associated with changes in outcomes outcomes to TTP. Further, no association was found between *TP53, PIK3CA* and *AKT1* mutations and TTP in HR-positive patients treated with endocrine therapy.

### Spectrum of *TP53* mutations

The majority of *TP53* mutations were missense mutations (72%) (Supplementary Table 2). In patients with TNBC, the relative proportion of nonsense and frameshift mutations was increased compared to HR-positive and HER2-positive patients.

## DISCUSSION

In this cohort of 500 MBC patients who underwent hotspot mutation testing, we describe the clinical characteristics and outcomes associated with the most frequently encountered mutations, *TP53, PIK3CA* and *AKT1*. Many large series report the incidence of these prevalent mutations in metastatic breast cancer patients, but few series correlate these mutations with clinical characteristics and outcomes in order to better understand their clinical impact. Patients in this cohort were evaluated for mutations in either the primary tumor, recurrent disease or both. As has been previously reported, when both primary and metastatic samples were analyzed, mutations between the two samples were generally concordant.[16]

Mutations in *TP53* and *PIK3CA* have previously been identified as of fundamental importance in cancer pathogenesis and resistance to therapy.[17-19]. The Cancer and Genome Atlas Project (TCGA) reported a high frequency of *TP53* (37%) and *PIK3CA* (36%) mutations in breast cancer patients.[11] *TP53* mutations were seen in the majority of basal-like cancers (80%), which is comprised mainly of TNBCs, and only in a minority of patients with Luminal A cancers (12%), which are strongly ER-positive tumors of low grade. Conversely, *PIK3CA* mutation was seen in 45% of Luminal A tumors, 39% of HER2-positive tumors and 9% of basal-like tumors. Similarly, sequencing of the METABRIC cohort revealed higher rates of *TP53* mutations in basal-like patients (65%) than Luminal A patients (9%).[20] We also found that that *TP53* mutation was more frequently encountered in TNBC patients, and *PIK3CA* mutation was more frequently encountered in HR-positive and HER2-positive patients. The incidence of *TP53* and *PIK3CA* mutation was comparatively lower in our cohort, and this is at least in part attributable to the platforms used in our study, as we assessed only hotspot mutations.

It is well established that the *TP53* tumor suppressor protein is frequently mutated in cancer.[21, 22] TP53 mutations have been found to confer a poor prognosis in several tumor types.[23-25] Most studies report that the *TP53* is mutated in about 30% of breast cancers. Despite this, the prognostic impact of *TP53* mutations across the different molecular subtypes of breast cancer is still poorly understood. This is at least in part due to the fact that *TP53* is a multi-functional protein, and mutations in different domains may impart different phenotypes that are biologically distinct.[12] While some studies have reported increased chemosensitivity in patients with *TP53* mutations,[26, 27] others have reported the opposite.[28] Interestingly, patients with *TP53* mutation had worse TTP in this cohort of patients, but it is unclear if this was due to the presence of *TP53* mutation or the prevalence of this mutation in TNBCs. Further, most studies of chemosensitivity have been done in the early disease setting, whereas patients in this study all had advanced disease. A recent study reported that *TP53* mutation was associated with worse outcomes overall and specifically in patients with HR-positive disease.[20] Similarly, in our cohort, *TP53* mutation remained a significant predictor of worse clinical outcomes after multivariable analysis in the overall cohort and HR-positive patients. Further, basal-like breast cancers have previously been associated with truncation mutations in *TP53*.[29] Similarly, there was a higher incidence of nonsense/frameshift *TP53* mutations in the TNBC subset compared to other subsets in this cohort. The predictive analysis of different TP53 mutations would be incomplete as we assessed only hotspot mutations and thus missed some of the *TP53* genomic alterations.

PI3K pathway aberrations, especially in the p110α catalytic domain of *PI3K* which is encoded by *PIK3CA*, are common in breast cancer.[30, 31] However, the impact of these aberrations on prognosis remains unclear. It has been demonstrated that PI3K pathway aberrations are inconsistently associated with activation of downstream signaling, indicating that different PI3K pathway aberrations are of variable significance.[32] Although most studies associate mutations in *PIK3CA* with improved outcomes in breast cancer, [13, 33] some studies associate them with worse outcomes.[15] This may be partly due to the significance of specific mutations; it has been suggested that mutations in exon 20 of *PIK3CA* (kinase domain) are associated with improved outcomes, while mutations in exon 9 (helical domain) are associated with worse outcomes.[34] Further, specific genome alterations of *PIK3CA* have been associated with worse outcomes.[35] Tumor evolution may also play a role as high levels of intra-tumor heterogeneity have been associated with worse outcomes.[35] Additionally, in patients with HR-positive and HER2-positive tumors, increased PI3K/AKT/mTOR signaling has been associated with resistance to endocrine and HER2-targeted therapies, respectively.[36-41] In this cohort of MBC patients, *PIK3CA* hotspot mutations were associated with better clinical outcomes. However, after multivariable analysis *PIK3CA* mutation did not remain a significant predictor of outcomes in the overall cohort or HR-positive patients. Thus, the association with outcomes may be due to the prevalence of *PIK3CA* mutations in HR-positive patients rather than an independent effect. Further, when mutations in exon 9 were compared to mutations in exon 20 in this cohort, no significant differences in outcomes were seen.

Although the number of matched primary and metastatic tumors were small, the data was notable for an overall concordance rate of 74%. There was discordance in potentially actionable genes such as *PIK3CA* with both gains and losses seen, with a slight trend towards enrichment for *TP53* mutations and mutations in the MAPK pathway in metastases/recurrences. The mutations detected only in metastatic pairs supports the clonal evolution pattern of development during tumor progression while the presence of mutations in the primary but not in the paired metastasis, suggests that metastatic tumor branched off from the primary before it acquired those mutations. This genomic evolution. suggests the role of biopsy of metastases for genomic testing. Further study is needed to evaluate impact of such genomic discordance on outcomes with genomically- targeted therapies, as well as circulating DNA mutation status in patients with mutation discordances between primary and metastases.

This study has several limitations. One of the greatest limitations of this study is that only hotspot mutations in specific genes of interest were evaluated. As a result, some of the aberrations present in the tumor samples remain unknown, and the impact of these aberrations on clinical outcomes is unclear. Further, hotspot testing limits association of specific aberrations with outcomes since the potential implications of other aberrations remain unknown. However, the increased cost of performing full sequencing prohibits such detailed evaluation of large cohorts of patients such as this one. Therefore, data presented here is hypothesis-generating, and conclusions need to be verified with more extensive sequencing data. Another limitation of this study is that samples were collected at various timepoints in treatment. Some tumor samples were collected prior to any treatment while others were collected after varying duration of treatment. It is well known that treatment can lead to selection of specific aberrations. Therefore, again, any data presented here would need to be further verified in a predictive, uniform patient analysis prior to clinical application. Finally, mutations predicted to be germline in this cohort were based on an informatics algorithm that assessed allelic frequency in tumor samples only. Therefore, mutations that were predicted to be germline but were actually somatic might have been excluded. Overall, the number of such mutations to be excluded should be small.

In summary, in this cohort of MBC patients, *TP53*, the most frequently encountered hotspot mutation, was associated with worse clinical outcomes, while *PIK3CA*, the second most frequently encountered hotspot mutation did not remain a significant predictor of outcomes after multivariable analysis. This study assesses the prognostic impact of common hotspot mutations in metastatic breast cancer patients. In order to incorporate these findings into clinical use, the predictive value of these findings needs to be established with more extensive sequencing analysis in a uniform clinical setting. Based on predictive models, biomarker-driven trials of genomically-informed therapy can be designed with novel targeted agents. Alternatively, these models could be used to predict sensitivity to currently used chemotherapeutic, hormonal or targeted agents to improve outcomes in breast cancer patients.

## MATERIALS AND METHODS

Patients with advanced disease who were felt to benefit from somatic genomic testing were enrolled on an Institutional Review Board-approved protocol at The University of Texas MD Anderson Cancer (NCT01772771). Tumor samples were obtained from 500 patients with metastatic breast cancer after informed consent. Clinical outcomes were collected by a retrospective review, supplementing a prospectively maintained database.

### Tumor samples

Testing was performed on archived tumor samples for most patients. Testing was requested on both the primary tumor and metastases if both samples were available. Specimens included formalin-fixed paraffin-embedded (FFPE) core needle biopsies and tumor resection specimens. Manual macrodissection of tumor-rich areas was performed, and only cases with >20% tumor cellularity were included in this study.

Histologic grading was done using the Nottingham grading system combining nuclear grade, tubule formation and mitotic rate and assigning a combined score of 1 (low grade), 2 (intermediate grade), and 3 (high grade). Immunohistochemical (IHC) staining for estrogen receptor (antibody clone 6F11-Novacastra) and progesterone receptor (antibody clone PgR1294-DAKO) was scored as positive if ≥ 1% of tumor cell nuclei were immunoreactive.[42] IHC for HER2 was performed using antibody AB8 (NeoMarkers), and equivocal or indeterminate cases were evaluated by fluorescent in situ hybridization (FISH). HER2 status was scored according to current practice guidelines.[43] All histologic diagnoses, grading and IHC staining were evaluated by the breast pathology service at MD Anderson Cancer Center. Breast cancer subtypes were defined as: HR-positive (estrogen or progesterone receptor-positive and HER2-negative); HER2-positive (HER2-positive regardless of HR status) and TNBC (HR-negative and HER2-negative).

### Genomic analysis

DNA was extracted and amplified using standard methods.[44] Initially, the Sequenom multiplex assay was used to assess for single nucleotide variants and small insertions/deletions in hotspot regions in 11 genes: *AKT1, BRAF, GNAS, GNAQ, IDH1, IDH2, KRAS, MET, NRAS, PIK3CA* and *RET*. The Sequenom multiplex assay is based on distinguishing allele-specific primer extension products by mass spectrometry.[45] Later in the study, a 46 or 50 gene Ampliseq Ion Torrent Assay was used. This panel is a multiplex PCR-based library preparation method by which 190 regions (70-150 bp) that encompass 740 mutational hotspots in the coding sequence of 46 or 50 cancer-related genes are selectively amplified and subsequently analyzed for mutations using multiple markers of detection.[46] The following genes were included in the 46 gene panel: ABL1, *AKT1, ALK, APC, ATM, BRAF, CDH1, CDKN2A, CSF1R, CTNNB1, EGFR, ERBB2, ERBB4, FBXW7, FGFR1, FGFR2, FGFR3, FLT3, GNAS, HNF1A, HRAS, IDH1, JAK2, JAK3, KDR, KIT, KRAS, MET, MLH1, MPL, NPM1, NOTCH1, NRAS, PDGFRA, PIK3CA, PTEN, PTPN11, RB1, RET, SMAD4, SMARCB1, SMO, SRC, STK11, TP53* and *VHL*.[47] The 50 gene panel also included *EZH2, GNA11, GNAQ* and *IDH2*.

### Clinical outcomes

All patients were included in genomic analysis to assess frequencies of encountered mutations, but patients were excluded from outcomes analysis if they did not start systemic therapy at the time of diagnosis of metastatic disease or if they developed contralateral breast cancer prior to metastatic disease as it was unclear which cancer had metastasized. Treatment in the metastatic setting was conducted according to physician's choice. Some patients were enrolled in clinical trials based on encountered mutations. Relapse-free survival (RFS) was calculated from the date of initial breast cancer diagnosis to the date of first local or distant relapse, death or last follow-up. Distant relapse-free survival (DRFS) was calculated from the date of breast cancer diagnosis to the date of distant relapse, death, or last follow-up. Patients who had Stage IV disease at diagnosis, DRFS < 3 months or did not have definitive surgery were excluded from RFS and DRFS analysis. Local-regional relapse-free survival (LRFS) was calculated from the date of definitive surgery to the date of first breast, chest wall, or ipsilateral nodal basin recurrence, death or last follow-up. Patients who developed distant recurrence prior to local recurrence were still included in LRFS analysis. However, only patients that underwent definitive surgery were included (*N* = 375). Overall survival (OS) was calculated from the date of diagnosis of distant disease to the date of death or last follow-up. Time to treatment failure due to progression (TTP) was calculated from the date of treatment start in the metastatic setting to date of treatment end due to progression. Patients that discontinued treatment due to completion of therapy, toxicity, loss to follow up or death were censored. For TTP analysis, patients were grouped by type of therapy (chemotherapy [given alone or in combination with other agents], hormonal therapy or targeted therapy [including HER2-directed agents, mTOR inhibitors and agents on clinical trial, given alone or in combination with hormonal therapy but not in combination with chemotherapy]) and line of therapy (first, second or third) in the metastatic setting.

### Statistical analysis

Continuous variables were summarized using the median (range). Categorical variables were summarized by frequency and percent. Mutation rates were calculated based on the tested samples. The association between mutation status and categorical patient characteristics was assessed by Fisher's exact test. *P* values < 0.05 were considered significant. The distribution of time to event endpoints were estimated by the method of Kaplan and Meier, and the comparison between groups was conducted by the log-rank test. Clinical variables which were significant or marginally significant (*P* < 0.1) in univariable analysis were considered for inclusion in multivariable models. Cox proportional hazards model was used to evaluate the association between known prognostic and predictive clinical variables and patient outcomes in the multivariable analysis.

## SUPPLEMENTARY MATERIALS TABLES AND FIGURES



## References

[1] Jemal A, Bray F, Center M, Ferlay J, Ward E, Forman D (2011). Global cancer statistics. CA Cancer J Clin.

[2] Ferlay J, Soerjomataram I, Ervik M, Dikshit R, Eser S, Mathers C, Rebelo M, Parkin D, Forman D, Bray F (2014). GLOBCAN 2012 v1.1, Cancer Incidence and Mortality Worldwide: IARC CancerBase No. 11.

[3] O'shaughnessy J (2005). Extending survival with chemotherapy in metastatic breast cancer. Oncologist.

[4] Chia S, Speers C, D'yachkova Y, Kang A, Malfair-Taylor S, Barnett J, Coldman A, Gelmon K, O'Reilly S, Olivotto I (2007). The impact of new chemotherapeutic and homone agents on survival in a population-based cohort of women with metastatic breast cancer. Cancer.

[5] Slamon D, Leyland-Jones B, Shak S, Fuchs H, Paton V, Bajamonde A, Fleming T (2001). Use of chemotherapy plus a monoclonal antibody against HER2 for metastatic breast cancer that overexpresses HER2. N Engl J Med.

[6] Baselga J, Campone M, Piccart M, Burris H, Rugo H, Sahmoud T (2012). Everolimus in postmenopausal hormore-receptor-positive advanced breast cancer. N Engl J Med.

[7] Bachelot T, Bourgier C, Cropet C, Ray-Coquard I, Ferrero J, Freyer G, Abadie-Locourtoisie S (2012). Randomized phase II trial of everolimus in combination with tamoxifen in patients with hormone receptor-positive, human epidermal growth factor receptor 2-negative metastatic breast cancer with prior exposure to aromatase inhibitors: A GINECO Study. J Clin Oncol.

[8] Finn R, Crown J, Lang I, Boer K, Bondarenko I, Kulyk S, Ettl J (2014). Final results of a randomized phase II study of PD 0332991, a cyclin-dependent kinase (CDK)-4/6 inhibitor in combination with letrozole vs letrozole alone for first-line treatment of ER+/HER2- advance breast cancer.

[9] Turner N, Jungsil R, Andre F, Loi S, Verma S, Iwata H (2015). Palbocilib in Hormone-Receptor-Positive Advanced Breast Cancer. N Engl J Med.

[10] Winer E, Gralow J, Diller L, Karlan B, Loehrer P, Pierce L, Demetri G (2009). Clinical cancer advances 2008: Major research advances in cancer treatment, prevention, and scrrening - a report from the American Society of Clinical Oncology. J Clin Oncol.

[11] Network TCGA (2012). Comprehensive molecular portraits of human breast tumours. Nature.

[12] Muller P, Vousden K (2014). Mutant p53 in cancer: new functions and therapeutic opportunities. Cancer Cell.

[13] Mukohara T (2015). PI3K mutations in breast cancer: prognostic and therapeutic implications. Breast Cancer.

[14] Perez-Tenorio G, Stal O, Southeast Sweden Breast Cancer G (2002). Activation of AKT/PKB in breast cancer predicts a worse outcome among endocrine treated patients. Br J Cancer.

[15] Li SY, Rong M, Grieu F, Lacopetta B (2006). PIK3CA mutations in breast cancer are associated with poor outcome. Breast Cancer Res Treat.

[16] Meric-Bernstam F, Frampton GM, Ferrer-Lozano J, Yelensky R, Perez-Fidalgo JA, Wang Y, Palmer GA, Ross JS, Miller VA, Su X, Eroles P, Barrera JA, Burgues O, Lluch AM, Zheng X, Sahin A (2014). Concordance of genomic alterations between primary and recurrent breast cancer. Mol Cancer Ther.

[17] Schiff R, Massarweh SA, Shou J, Bharwani L, Mohsin SK, Osborne CK (2004). Cross-talk between estrogen receptor and growth factor pathways as a molecular target for overcoming endocrine resistance. Clin Cancer Res.

[18] Ali S, Coombes R (2002). Endocrine-responsive breast cancer and strategies for combating resistance. Nat Rev Cancer.

[19] Gajria D, Chandarlapaty S (2011). HER2-amplified breast cancer: mechanisms of trastuzumab resistance and novel targeted therapies. Expert Rev Anticancer Ther.

[20] Silwal-Pandit L, Vollan H, Chin S-F, Rueda O, McKinney S, Osako T, Quigley D (2014). TP53 mutation spectrum in breast cancer is subtype specific and has distinct prognostic relevance. Clin Cancer Res.

[21] Hollstein M, Sidransky D, Vogelstein B, Harris C (1991). p53 mutations in human cancers. Science.

[22] Levine A, Momand J, Finlay C (1991). The p53 tumour suppressor gene. Nature.

[23] Robles AI, Jen J, Harris CC (2016). Clinical Outcomes of TP53 Mutations in Cancers. Cold Spring Harb Perspect Med.

[24] Cleary SP, Jeck WR, Zhao X, Chen K, Selitsky SR, Savich GL, Tan TX, Wu MC, Getz G, Lawrence MS, Parker JS, Li J, Powers S, Kim H, Fischer S, Guindi M (2013). Identification of driver genes in hepatocellular carcinoma by exome sequencing. Hepatology.

[25] Churi CR, Shroff R, Wang Y, Rashid A, Kang HC, Weatherly J, Zuo M, Zinner R, Hong D, Meric-Bernstam F, Janku F, Crane CH, Mishra L, Vauthey JN, Wolff RA, Mills G (2014). Mutation profiling in cholangiocarcinoma: prognostic and therapeutic implications. PLoS One.

[26] Bertheau P, Plassa F, Espie M, Turpin E, de Roquancourt A, Marty M (2002). Effect of mutated TP53 on response of advanced breast cancers to high-dose chemotherapy. Lancet.

[27] Kandioler-Eckersberger D, Ludwig C, Rudas M, Kappel S, Janschek E, Wenzel C, Schlagbauer-Wadl H (2000). TP53 mutation and p53 overexpression for prediction of response to neoadjuvant treatment in breast cancer patients. Clin Cancer Res.

[28] Geisler S, Borresen-Dale A, Johnsen H, Aas T, Geisler J, Akslen L (2003). TP53 gene mutations predict the response to neoadjuvant treatment with 5-fluorouracil and mitomycin in locally advanced breast cancer. Clin Cancer Res.

[29] Holstege H, Horlings H, Velds A, Langerod A, Borresen-Dale A, van de Vijver M (2010). BRCA1-mutated and basal-like breast cancers have simial aCGH profiles and a high incidence of protein truncating TP53 mutations. BMC Cancer.

[30] Campbell IG, Russell SE, Choong DY, Montgomery KG, Ciavarella ML, Hooi CS, Cristiano BE, Pearson RB, Phillips WA (2004). Mutation of the PIK3CA gene in ovarian and breast cancer. Cancer Res.

[31] Bachman KE, Argani P, Samuels Y, Silliman N, Ptak J, Szabo S, Konishi H, Karakas B, Blair BG, Lin C, Peters BA, Velculescu VE, Park BH (2004). The PIK3CA gene is mutated with high frequency in human breast cancers. Cancer Biol Ther.

[32] Stemke-Hale K, Gonzalez-Angulo AM, Lluch A, Neve RM, Kuo WL, Davies M, Carey M, Hu Z, Guan Y, Sahin A, Symmans WF, Pusztai L, Nolden LK, Horlings H, Berns K, Hung MC (2008). An integrative genomic and proteomic analysis of PIK3CA, PTEN, and AKT mutations in breast cancer. Cancer Res.

[33] Perez-Tenorio G, Alkhori L, Olsson B, Waltersson MA, Nordenskjold B, Rutqvist LE, Skoog L, Stal O (2007). PIK3CA mutations and PTEN loss correlate with similar prognostic factors and are not mutually exclusive in breast cancer. Clin Cancer Res.

[34] Barbareschi M, Buttitta F, Felicioni L, Cotrupi S, Barassi F, Del Grammastro M, Ferro A, Dalla Palma P, Galligioni E, Marchetti A (2007). Different prognostic roles of mutations in the helical and kinase domains of the PIK3CA gene in breast carcinomas. Clin Cancer Res.

[35] Pereira B, Chin SF, Rueda OM, Vollan HK, Provenzano E, Bardwell HA, Pugh M, Jones L, Russell R, Sammut SJ, Tsui DW, Liu B, Dawson SJ, Abraham J, Northen H, Peden JF (2016). The somatic mutation profiles of 2,433 breast cancers refines their genomic and transcriptomic landscapes. Nat Commun.

[36] Perez-Tenorio G, Stal O (2002). Activation of AKT/PKB in breast cancer patients predicts a worse outcome among endocrine treated patients. Br J Cancer.

[37] Miller T, Perez-Torres M, Narasanna A, Guix M, Stal O, Tenorio GP (2009). Loss of Phosphatase and Tensin homologue deleted on chromosome 10 engasges ErbB3 and insulin-like growth factor-I receptor signaling to promote antiestrogen resistance in breast cancer. Cancer Res.

[38] Nagata Y, Lan K, Zhou X, Tan M, Esteva F, Sahin A, Klos K (2004). PTEN activation contributes to tumor inhibition by trastuzumab, and loss of PTEN predicts trastuzumab resistance in patients. Cancer Cell.

[39] Berns K, Horlings H, Hennessy B, Madiredjo M, Hijmans E, Beelen K, Linn S (2007). A functional genetic approach identifies the PI3K pathway as a major determinant of trastuzumab resistance in breast cancer. Cancer Cell.

[40] Baselga J, Cortes J, Im S, Clark E, Ross G, Kiermaier A, Swain S (2014). Biomarker analyses in CLEOPATRA: a phase III, placebo-controlled study of pertuzumab in human epidermal growth factor receptor 2-positive, first-line metastatic breast cancer. J Clin Oncol.

[41] Loibl S, Minckwitz GV, Schneeweiss A, Paepke S (2014). PIK3CA mutations are associated with lower rates of pathologic complete response to anti-human epidermal growth factor receptor 2 (her2) therapy in primary HER2-overexpressing breast cancer. J Clin Oncol.

[42] Hammond M, Hayes D, Dowsett M, Allred D, Hagerty K, Badve S (2010). American Society of Clinical Oncology/College of American Pathologists guideline recommendations for immunohistochemical testing of estrogen and progesterone receptors in breast cancer. Arch Pathol Lab Med.

[43] Wolff A, Hammond M, Hicks D, Dowsett M, McShane L, Allison K, Allred D, Bartlett J (2014). Recommendations for human epidermal growth factor receptor 2 testing in breast cancer: American Society of Clinical Oncology/College of American Pathologists clinical practice. Arch Pathol Lab Med.

[44] Singh RR PK, Routbort MJ, Reddy NG, Barkoh BA, Handal B (2013). Clinical Validation of a next-generation sequencing screen for mutational hotspots in 46 cancer-related genes. J Mol Diagn.

[45] Bradic M, Costa J, Chelo IM (2011). Genotyping with Sequenom. Methods Mol Biol.

[46] Singh RR, Patel KP, Routbort MJ, Reddy NG, Barkoh BA, Handal B, Kanagal-Shamanna R, Greaves WO, Medeiros LJ, Aldape KD, Luthra R (2013). Clinical validation of a next-generation sequencing screen for mutational hotspots in 46 cancer-related genes. J Mol Diagn.

[47] Kanagal-Shamanna R, Portier B, Singh R, Routbort M, Aldape K, Handal B, Rahimi H, Reddy N (2014). Next-generation sequencing-based multi-gene mutation profiling of solid tumors using fine needle aspiration samples: promises and challenges for routine clinical diagnostics. Mod Pathol.

