# Supplementary material for: Clinical outcomes based on multigene profiling in metastatic breast cancer patients
Source: Oncotarget. 2016 Oct 28;7(47):76362–73. doi: 10.18632/oncotarget.12987 (PMC5363515; doi:10.18632/oncotarget.12987)
Supplement: Supplementary file 1 [file oncotarget-07-76362-s001.pdf]

Clinical outcomes based on multigene profiling in metastatic breast cancer patients

Supplementary Material

Supplementary Table 1: List of the genes tested in Sequenom, CMS 46 and CMS 50 gene assays

Sequenom	CMS 46	CMS 50
AKT1	*ABL1*	*ABL1*
BRAF	*AKT1*	*AKT1*
GNAQ	*ALK*	*ALK*
GNAS	*APC*	*APC*
IDH1	*ATM*	*ATM*
IDH2	*BRAF*	*BRAF*
KRAS	*CDH1*	*CDH1*
MET	*CDKN2A*	*CDKN2A*
NRAS	*CSF1R*	*CSF1R*
PIK3CA	*CTNNB1*	*CTNNB1*
RET	*EGFR*	*EGFR*
	ERBB2	*ERBB2*
	ERBB4	*ERBB4*
	FBXW7	*EZH2*
	FGFR1	*FBXW7*
	FGFR2	*FGFR1*
	FGFR3	*FGFR2*
	FLT3	*FGFR3*
	GNAS	*FLT3*
	HNF1A	*GNA11*
	HRAS	*GNAQ*

	IDH1	*GNAS*
	JAK2	*HNF1A*
	JAK3	*HRAS*
	KDR	*IDH1*

Supplementary Table 1 (continued). List of the genes tested in Sequenom, CMS 46 and CMS 50 gene assays

Sequenom	CMS 46	CMS 50
	KIT	*IDH2*
	KRAS	*JAK2*
	MET	*JAK3*
	MLH1	*KDR*
	MPL	*KIT*
	NOTCH1	*KRAS*
	NPM1	*MET*
	NRAS	*MLH1*
	PDGFRA	*MPL*
	PIK3CA	*NOTCH1*
	PTEN	*NPM1*
	PTPN11	*NRAS*
	RB1	*PDGFRA*
	RET	*PIK3CA*
	SMAD4	*PTEN*
	SMARCB1	*PTPN11*
	SMO	*RB1*
	SRC	*RET*
	STK11	*SMAD4*
	TP53	*SMARCB1*
	VHL	*SMO*

SRC
STK11
TP53
VHL

Supplementary Table 2: List of Potential Germline Variants

Gene	Codon	WildType	VariantType
ABL1	**247**	**K**	**R**
APC	**870**	**P**	**S**
APC	**1317**	**E**	**Q**
ATM	**410**	**V**	**A**
ATM	**604**	**P**	**S**
ATM	**858**	**F**	**L**
ATM	**1309**	**A**	**T**
ATM	**1691**	**S**	**R**
FGFR3	**384**	**F**	**L**
JAK3	**132**	**P**	**T**
JAK3	**722**	**V**	**I**
KDR	**482**	**C**	**R**
KDR	**1356**	**V**	**A**
KIT	**541**	**M**	**L**
MET	**168**	**E**	**D**
MET	**362**	**M**	**T**
MET	**375**	**N**	**S**
MET	**1010**	**T**	**I**
MLH1	**384**	**V**	**D**
PIK3CA	**391**	**I**	**M**
STK11	**354**	**F**	**L**

TP53	**273**	**R**	**H**

Supplementary Table 3: Functional Class of *TP53* Mutations

Class	Overall	HR-Positive	TNBC	HER2-Positive
**Missense**	87 (72%)	48 (76%)	27 (61%)	12 (86%)
**Nonsense/Frameshift**	34 (28%)	15 (24%)	17 (39%)	2 (14%)

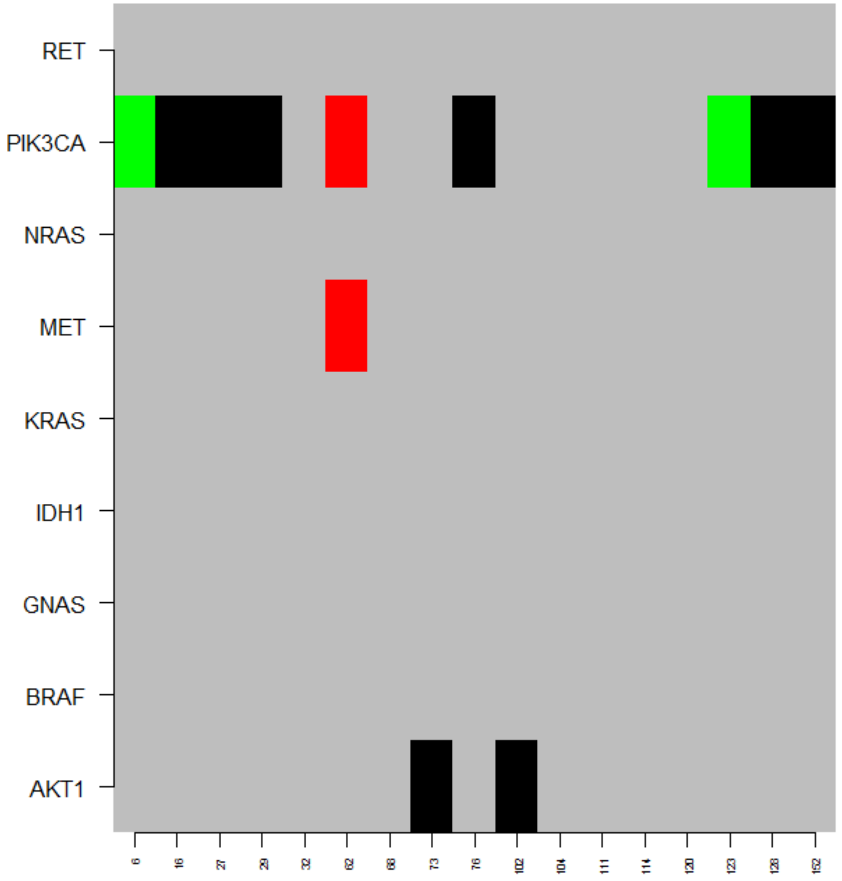

Supplementary Figure 1: **Concordance of mutations analyzed by Sequenom and Ampliseq 46 gene assays.** Mutation status was analyzed for the overlapping genes in 17 patients who underwent analysis by both Sequenom and Ampliseq 46 gene assays. Mutation status of the genes in each testing assay is indicated by color: Gray: wild type by both Sequenom and Ampliseq 46; Black: mutated by both Sequenom and Ampliseq 46; Red: wild type by Sequenom and mutated by Ampliseq 46; Green: mutated by Sequenom and wild type by Ampliseq 46.

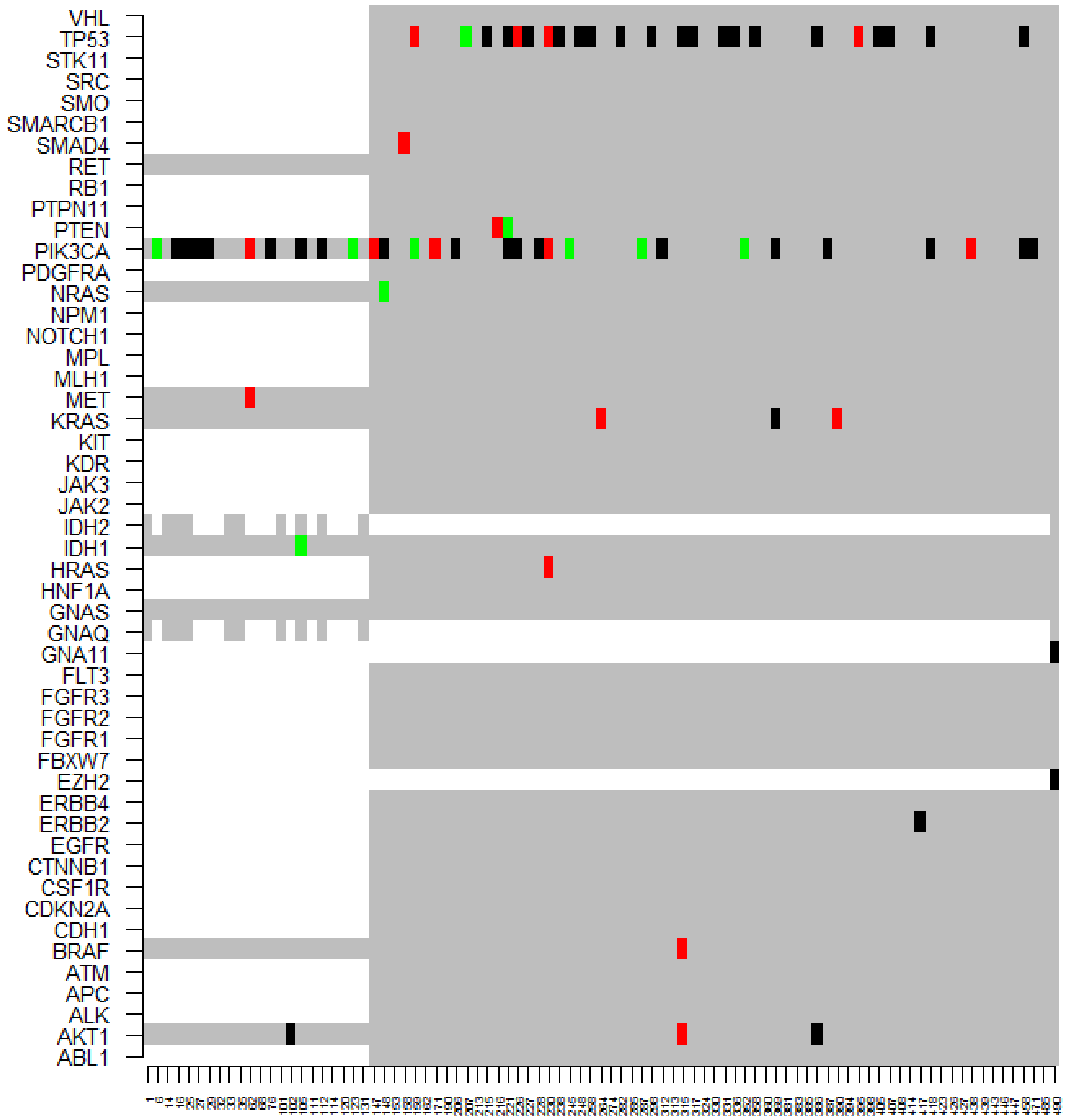

Supplementary Figure 2: **Concordance of mutations detected in primary and metastatic tumors.** Analysis was performed for concordance in 89 patients who had both primary tumors and metastases analyzed. Mutation status of the genes in each patient is indicated by color: White: null in both primary and metastatic samples; Gray: wild type in both primary and metastatic samples; Black: mutated in both primary and metastatic samples; Red: wild type in primary and mutated in metastatic sample; Green: mutated in primary and wild type in metastatic sample.

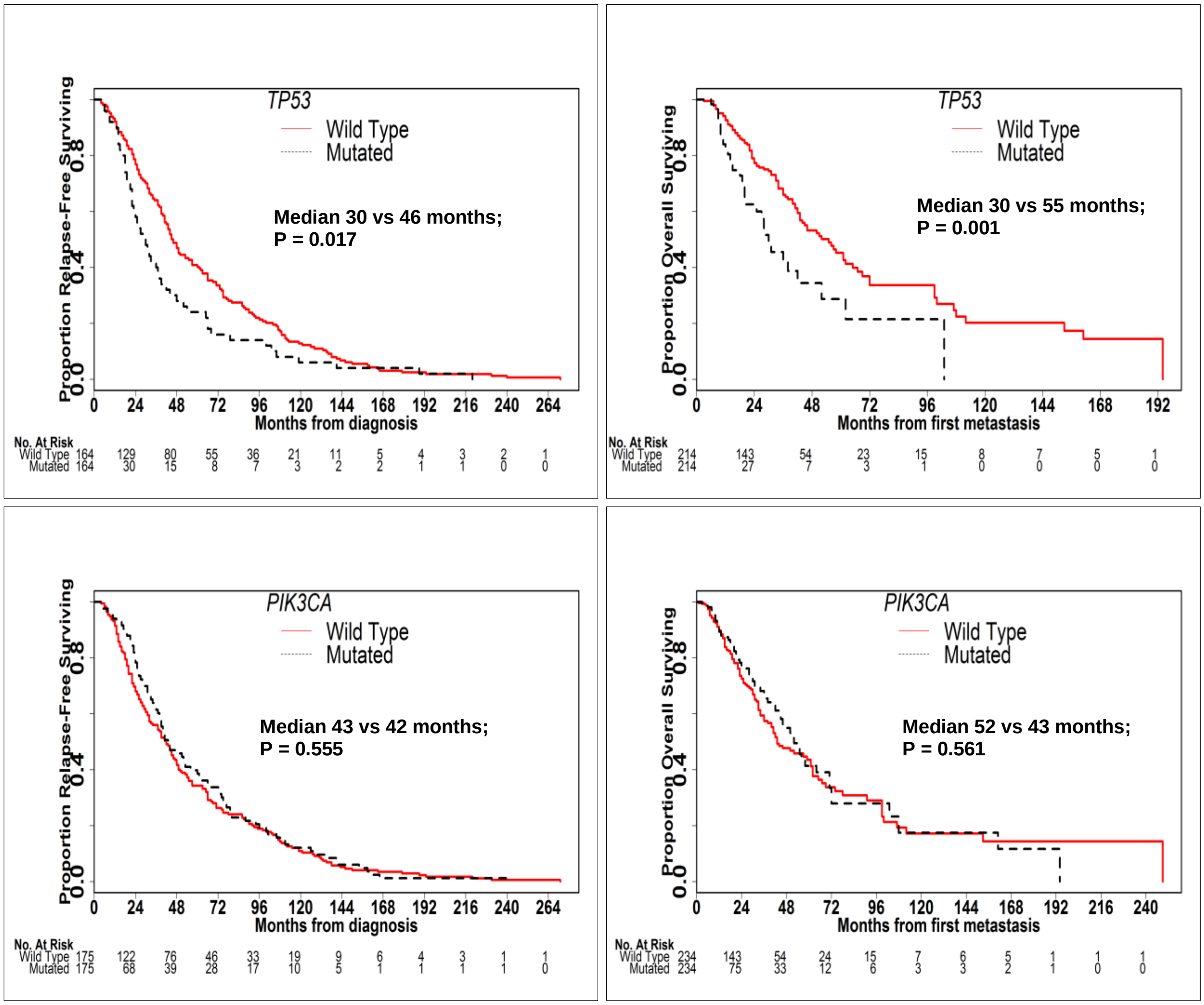

Supplementary Figure 3: RFS and OS in HR-Positive Patients with *TP53* or *PIK3CA* Mutation

Kaplan-Meier curves of RFS and OS in HR-positive patients with and without *TP53* and *PIK3CA* mutations.

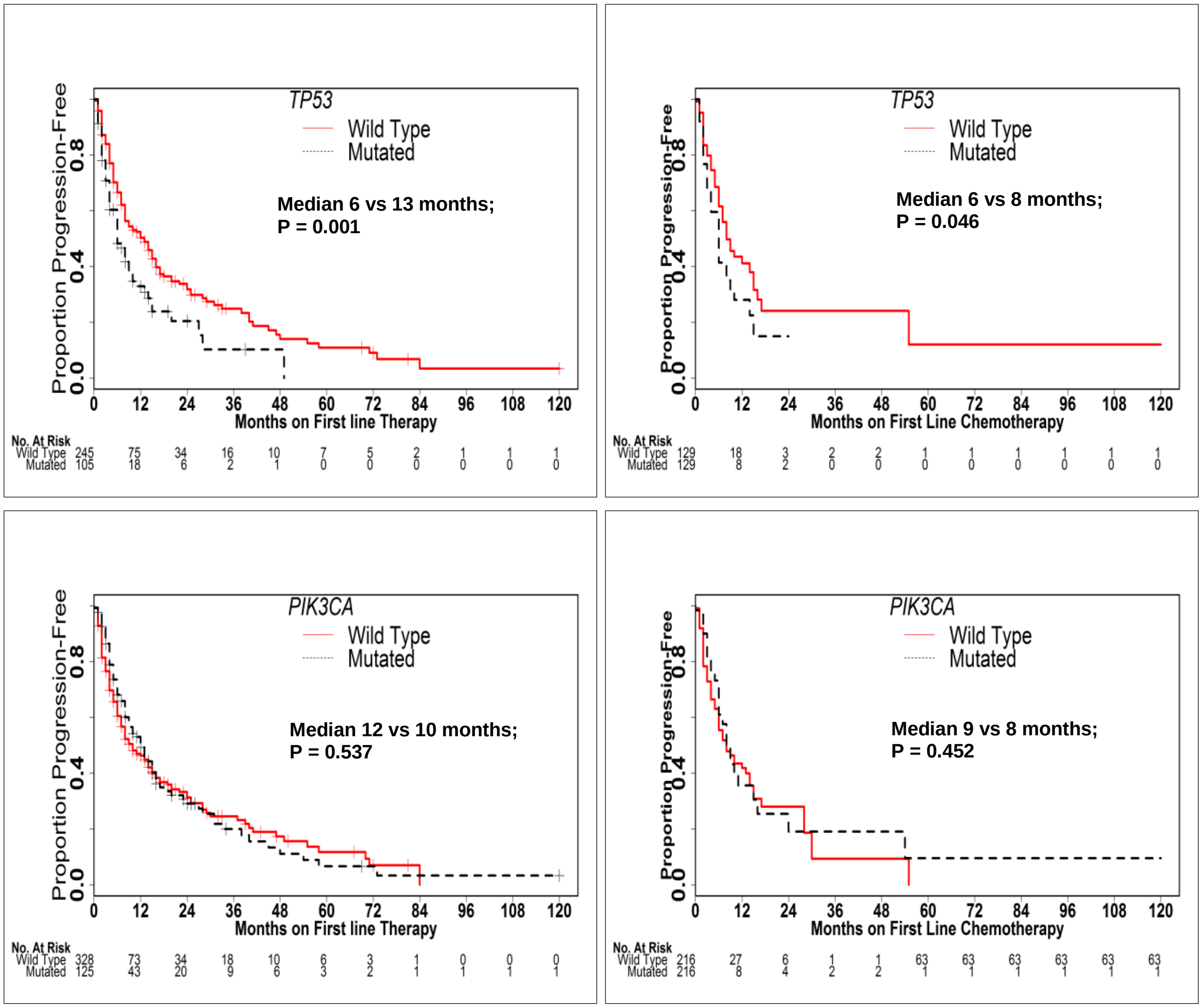

Supplementary Figure 4: TTP in Patients with *TP53* or *PIK3CA* Mutation

Kaplan-Meier curves of TTP with first line therapy and first line chemotherapy in the metastatic setting for patients with and without *TP53* and *PIK3CA* mutations.
